# *N*-(2-Pyrid­yl)-4-toluidine

**DOI:** 10.1107/S1600536808037306

**Published:** 2008-11-26

**Authors:** Zainal Abidin Fairuz, Zaharah Aiyub, Zanariah Abdullah, Seik Weng Ng

**Affiliations:** a Department of Chemistry, University of Malaya, 50603 Kuala Lumpur, Malaysia

## Abstract

There are two mol­ecules in the asymmetric unit of the title compound, C_12_H_12_N_2_, with dihedral angles between the aromatic rings of 48.35 (12) and 51.02 (12)°. In the crystal structure, both mol­ecules form inversion dimers, linked by pairs of N—H⋯N hydrogen bonds.

## Related literature

For the crystal structure of *N*-(2-pyrid­yl)aniline, see: Polamo *et al.* (1997 ▶)
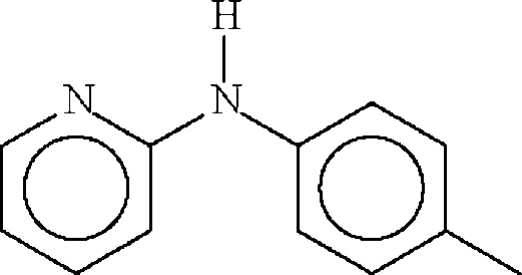


## Experimental

### 

#### Crystal data

C_12_H_12_N_2_
*M* *_r_* = 184.24
Monoclinic, 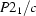

*a* = 18.2260 (7) Å
*b* = 10.5680 (3) Å
*c* = 10.6005 (3) Å
β = 95.364 (2)°
*V* = 2032.9 (1) Å^3^
*Z* = 8
Mo *K*α radiation
μ = 0.07 mm^−1^
*T* = 100 (2) K
0.30 × 0.10 × 0.05 mm

#### Data collection

Bruker SMART APEX diffractometer
Absorption correction: none
18430 measured reflections
4676 independent reflections
2774 reflections with *I* > 2σ(*I*)
*R*_int_ = 0.066

#### Refinement

*R*[*F* ^2^ > 2σ(*F* ^2^)] = 0.070
*wR*(*F* ^2^) = 0.206
*S* = 1.01
4676 reflections
263 parameters
2 restraints
H atoms treated by a mixture of independent and constrained refinement
Δρ_max_ = 0.64 e Å^−3^
Δρ_min_ = −0.31 e Å^−3^

### 

Data collection: *APEX2* (Bruker, 2007 ▶); cell refinement: *SAINT* (Bruker, 2007 ▶); data reduction: *SAINT*; program(s) used to solve structure: *SHELXS97* (Sheldrick, 2008 ▶); program(s) used to refine structure: *SHELXL97* (Sheldrick, 2008 ▶); molecular graphics: *X-SEED* (Barbour, 2001 ▶); software used to prepare material for publication: *publCIF* (Westrip, 2008 ▶).

## Supplementary Material

Crystal structure: contains datablocks global, I. DOI: 10.1107/S1600536808037306/sg2278sup1.cif

Structure factors: contains datablocks I. DOI: 10.1107/S1600536808037306/sg2278Isup2.hkl

Additional supplementary materials:  crystallographic information; 3D view; checkCIF report

## Figures and Tables

**Table 1:** Hydrogen-bond geometry (Å, °)

*D*—H⋯*A*	*D*—H	H⋯*A*	*D*⋯*A*	*D*—H⋯*A*
N1—H1n⋯N2^i^	0.88 (1)	2.06 (1)	2.944 (3)	174 (3)
N3—H3n⋯N4^ii^	0.91 (1)	2.08 (2)	2.949 (3)	159 (3)

Symmetry codes: (i) 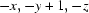; (ii) 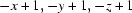.

**Fig. 1.**
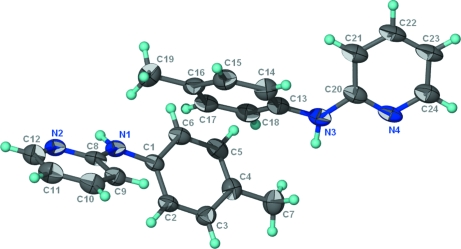
The molecular structure of (I), with displacement ellipsoids at the 70% probability level; hydrogen atoms are drawn as spheres of arbitrary radius.

## supplementary crystallographic information

### Comment

There are two molecules in the asymmetric unit (Fig. 1) of the title compound, C_12_H_12_N_2_, with dihedral angles between the aromatic rings of 48.35 (12)° and 51.02 (12)°. In the crystal, both molecules form inversion dimers, linked by pairs of N—H···N hydrogen bonds (Table 1).  For the related crystal structure of *N*-(2-pyridyl)aniline, see: Polamo *et al.* (1997)

### Experimental

Chloropyridine (0.5 ml, 0.5 mmol) and 4-toluidine (0.6 g, 0.5 mmol) were heated at 423–433 K for 3 h. The solid was dissolved in water. The compound was extracted with ether. The ether extract was dried over sodium sulfate. The solvent was evaporated and the product recrystallized from ethanol to yield colorless prisms of (I) among some unidentified dark brown materials.

### Refinement

The carbon-bound H-atoms were placed in calculated positions (C—H = 0.95–0.98 Å) and refined as riding with *U*(H) = 1.2–1.5*U*(C). The amino H-atom was located in a difference map, and was refined with a distance restraint of N–H 0.88±0.01 Å.

The highest difference peak is 1.0Å from C18 and deepest difference hole is 0.7Å from C18.

### Crystal data

**Table:**

C_12_H_12_N_2_	*F*_000_ = 784
*M**_r_* = 184.24	*D*_x_ = 1.204 Mg m^−^^3^
Monoclinic, *P*2_1_/*c*	Mo *K*α radiation λ = 0.71073 Å
Hall symbol: -P 2ybc	Cell parameters from 1819 reflections
*a* = 18.2260 (7) Å	θ = 2.2–24.4º
*b* = 10.5680 (3) Å	µ = 0.07 mm^−^^1^
*c* = 10.6005 (3) Å	*T* = 100 (2) K
β = 95.364 (2)º	Prism, colorless
*V* = 2032.9 (1) Å^3^	0.30 × 0.10 × 0.05 mm
*Z* = 8	

### Data collection

**Table:**

Bruker SMART APEX diffractometer	2774 reflections with *I* > 2σ(*I*)
Radiation source: fine-focus sealed tube	*R*_int_ = 0.066
Monochromator: graphite	θ_max_ = 27.5º
*T* = 100(2) K	θ_min_ = 1.1º
ω scans	*h* = −23→23
Absorption correction: None	*k* = −13→13
18430 measured reflections	*l* = −13→13
4676 independent reflections	

### Refinement

**Table:**

Refinement on *F*^2^	Secondary atom site location: difference Fourier map
Least-squares matrix: full	Hydrogen site location: inferred from neighbouring sites
*R*[*F*^2^ > 2σ(*F*^2^)] = 0.070	H atoms treated by a mixture of independent and constrained refinement
*wR*(*F*^2^) = 0.206	*w* = 1/[σ^2^(*F*_o_^2^) + (0.0922*P*)^2^ + 1.1608*P*] where *P* = (*F*_o_^2^ + 2*F*_c_^2^)/3
*S* = 1.01	(Δ/σ)_max_ = 0.001
4676 reflections	Δρ_max_ = 0.64 e Å^−^^3^
263 parameters	Δρ_min_ = −0.31 e Å^−^^3^
2 restraints	Extinction correction: none
Primary atom site location: structure-invariant direct methods	

### Fractional atomic coordinates and isotropic or equivalent isotropic displacement parameters (Å^2^)

**Table:**

	*x*	*y*	*z*	*U*_iso_*/*U*_eq_	
N1	0.09908 (12)	0.46043 (18)	0.0668 (2)	0.0267 (5)	
N2	−0.01070 (12)	0.37392 (18)	0.11163 (19)	0.0279 (5)	
N3	0.40746 (13)	0.5526 (2)	0.5578 (2)	0.0350 (6)	
N4	0.52478 (13)	0.60957 (18)	0.62565 (19)	0.0317 (5)	
C1	0.17505 (14)	0.4657 (2)	0.0530 (2)	0.0258 (5)	
C2	0.21791 (14)	0.3582 (2)	0.0393 (2)	0.0293 (6)	
H2	0.1962	0.2766	0.0418	0.035*	
C3	0.29141 (14)	0.3698 (3)	0.0221 (3)	0.0337 (6)	
H3	0.3198	0.2954	0.0138	0.040*	
C4	0.32560 (15)	0.4877 (3)	0.0164 (3)	0.0334 (6)	
C5	0.28179 (15)	0.5943 (2)	0.0269 (3)	0.0340 (6)	
H5	0.3031	0.6759	0.0208	0.041*	
C6	0.20817 (15)	0.5841 (2)	0.0460 (2)	0.0312 (6)	
H0	0.1798	0.6585	0.0543	0.037*	
C7	0.40666 (16)	0.4993 (3)	0.0012 (3)	0.0419 (7)	
H7A	0.4166	0.5822	−0.0350	0.063*	
H7B	0.4213	0.4325	−0.0555	0.063*	
H7C	0.4348	0.4907	0.0841	0.063*	
C8	0.06324 (14)	0.3732 (2)	0.1354 (2)	0.0269 (6)	
C9	0.09904 (15)	0.2931 (2)	0.2268 (2)	0.0311 (6)	
H9	0.1512	0.2944	0.2431	0.037*	
C10	0.05715 (17)	0.2127 (2)	0.2923 (3)	0.0371 (7)	
H10	0.0804	0.1573	0.3544	0.045*	
C11	−0.01855 (16)	0.2121 (2)	0.2684 (3)	0.0377 (7)	
H11	−0.0482	0.1566	0.3126	0.045*	
C12	−0.04948 (16)	0.2946 (2)	0.1783 (3)	0.0340 (6)	
H12	−0.1016	0.2955	0.1624	0.041*	
C13	0.32986 (15)	0.5449 (2)	0.5522 (3)	0.0324 (6)	
C14	0.29295 (16)	0.5298 (3)	0.6599 (3)	0.0377 (7)	
H14	0.3199	0.5265	0.7410	0.045*	
C15	0.21749 (16)	0.5196 (3)	0.6495 (3)	0.0379 (7)	
H15	0.1931	0.5108	0.7244	0.045*	
C16	0.17531 (15)	0.5217 (2)	0.5326 (3)	0.0342 (6)	
C17	0.21226 (16)	0.5333 (2)	0.4247 (3)	0.0343 (6)	
H17	0.1853	0.5331	0.3436	0.041*	
C18	0.28849 (16)	0.5451 (2)	0.4344 (3)	0.0357 (7)	
H18	0.3129	0.5536	0.3595	0.043*	
C19	0.09289 (16)	0.5105 (3)	0.5244 (3)	0.0446 (7)	
H19A	0.0753	0.4663	0.4461	0.067*	
H19B	0.0710	0.5951	0.5244	0.067*	
H19C	0.0786	0.4626	0.5975	0.067*	
C20	0.45389 (16)	0.6270 (2)	0.6362 (2)	0.0318 (6)	
C21	0.42898 (18)	0.7175 (2)	0.7187 (3)	0.0388 (7)	
H21	0.3779	0.7288	0.7261	0.047*	
C22	0.48103 (17)	0.7898 (2)	0.7891 (3)	0.0391 (7)	
H22	0.4653	0.8516	0.8458	0.047*	
C23	0.55493 (18)	0.7741 (2)	0.7787 (3)	0.0412 (7)	
H23	0.5908	0.8242	0.8264	0.049*	
C24	0.57493 (18)	0.6821 (2)	0.6957 (3)	0.0404 (7)	
H24	0.6258	0.6690	0.6874	0.049*	
H1N	0.0699 (14)	0.510 (2)	0.017 (2)	0.047 (9)*	
H3N	0.4165 (16)	0.495 (2)	0.498 (2)	0.045 (9)*	

### Atomic displacement parameters (Å^2^)

**Table:**

	*U*^11^	*U*^22^	*U*^33^	*U*^12^	*U*^13^	*U*^23^
N1	0.0326 (12)	0.0186 (10)	0.0293 (12)	0.0017 (9)	0.0053 (9)	0.0041 (8)
N2	0.0344 (12)	0.0213 (10)	0.0291 (12)	0.0011 (9)	0.0080 (9)	0.0022 (8)
N3	0.0438 (14)	0.0293 (12)	0.0327 (13)	−0.0009 (10)	0.0075 (11)	−0.0068 (10)
N4	0.0537 (15)	0.0173 (10)	0.0240 (11)	−0.0013 (10)	0.0032 (10)	−0.0002 (8)
C1	0.0341 (14)	0.0227 (12)	0.0206 (12)	−0.0018 (10)	0.0022 (10)	−0.0012 (9)
C2	0.0361 (15)	0.0211 (12)	0.0302 (14)	−0.0005 (11)	0.0012 (11)	−0.0006 (10)
C3	0.0340 (15)	0.0325 (14)	0.0342 (15)	0.0038 (12)	0.0006 (12)	0.0000 (11)
C4	0.0347 (15)	0.0372 (15)	0.0279 (14)	−0.0031 (12)	0.0015 (11)	0.0026 (11)
C5	0.0437 (17)	0.0266 (13)	0.0316 (15)	−0.0074 (12)	0.0022 (12)	0.0034 (11)
C6	0.0413 (16)	0.0220 (12)	0.0306 (14)	−0.0002 (11)	0.0043 (12)	0.0015 (10)
C7	0.0378 (17)	0.0472 (17)	0.0410 (17)	−0.0038 (13)	0.0053 (13)	0.0045 (13)
C8	0.0414 (15)	0.0165 (11)	0.0237 (13)	−0.0001 (10)	0.0077 (11)	−0.0019 (9)
C9	0.0399 (16)	0.0256 (13)	0.0273 (14)	−0.0003 (11)	0.0009 (11)	0.0009 (10)
C10	0.0548 (19)	0.0287 (14)	0.0280 (15)	−0.0006 (13)	0.0046 (13)	0.0070 (11)
C11	0.0512 (18)	0.0289 (14)	0.0342 (16)	−0.0050 (13)	0.0112 (13)	0.0072 (11)
C12	0.0413 (16)	0.0302 (14)	0.0320 (15)	−0.0034 (12)	0.0103 (12)	0.0034 (11)
C13	0.0402 (16)	0.0240 (13)	0.0331 (15)	0.0023 (11)	0.0033 (12)	−0.0010 (11)
C14	0.0434 (17)	0.0361 (15)	0.0338 (15)	0.0017 (13)	0.0043 (13)	0.0060 (12)
C15	0.0438 (17)	0.0321 (14)	0.0383 (16)	0.0028 (12)	0.0069 (13)	0.0044 (12)
C16	0.0366 (16)	0.0218 (12)	0.0449 (17)	0.0031 (11)	0.0083 (13)	−0.0004 (11)
C17	0.0455 (17)	0.0228 (13)	0.0350 (15)	0.0008 (12)	0.0056 (12)	0.0003 (11)
C18	0.0477 (17)	0.0266 (14)	0.0343 (15)	−0.0010 (12)	0.0112 (13)	−0.0005 (11)
C19	0.0379 (17)	0.0482 (18)	0.0481 (19)	0.0053 (14)	0.0067 (14)	0.0015 (14)
C20	0.0491 (17)	0.0213 (12)	0.0253 (14)	−0.0014 (11)	0.0044 (12)	0.0031 (10)
C21	0.0591 (19)	0.0267 (14)	0.0309 (15)	−0.0007 (13)	0.0052 (13)	−0.0021 (11)
C22	0.065 (2)	0.0221 (13)	0.0298 (15)	0.0027 (13)	0.0000 (14)	−0.0040 (11)
C23	0.069 (2)	0.0218 (13)	0.0313 (16)	0.0038 (13)	−0.0040 (14)	−0.0044 (11)
C24	0.0553 (19)	0.0253 (13)	0.0404 (17)	0.0010 (13)	0.0025 (14)	−0.0005 (12)

### Geometric parameters (Å, °)

**Table:**

N1—C8	1.376 (3)	C10—C11	1.379 (4)
N1—C1	1.407 (3)	C10—H10	0.9500
N1—H1N	0.884 (10)	C11—C12	1.375 (4)
N2—C12	1.340 (3)	C11—H11	0.9500
N2—C8	1.348 (3)	C12—H12	0.9500
N3—C20	1.376 (3)	C13—C14	1.388 (4)
N3—C13	1.412 (3)	C13—C18	1.398 (4)
N3—H3N	0.911 (10)	C14—C15	1.374 (4)
N4—C20	1.320 (3)	C14—H14	0.9500
N4—C24	1.359 (3)	C15—C16	1.396 (4)
C1—C2	1.393 (3)	C15—H15	0.9500
C1—C6	1.395 (3)	C16—C17	1.385 (4)
C2—C3	1.374 (4)	C16—C19	1.501 (4)
C2—H2	0.9500	C17—C18	1.389 (4)
C3—C4	1.397 (4)	C17—H17	0.9500
C3—H3	0.9500	C18—H18	0.9500
C4—C5	1.391 (4)	C19—H19A	0.9800
C4—C7	1.506 (4)	C19—H19B	0.9800
C5—C6	1.380 (4)	C19—H19C	0.9800
C5—H5	0.9500	C20—C21	1.400 (4)
C6—H0	0.9500	C21—C22	1.381 (4)
C7—H7A	0.9800	C21—H21	0.9500
C7—H7B	0.9800	C22—C23	1.372 (4)
C7—H7C	0.9800	C22—H22	0.9500
C8—C9	1.400 (3)	C23—C24	1.382 (4)
C9—C10	1.374 (4)	C23—H23	0.9500
C9—H9	0.9500	C24—H24	0.9500
			
C8—N1—C1	127.0 (2)	C10—C11—H11	121.2
C8—N1—H1N	115 (2)	N2—C12—C11	124.1 (3)
C1—N1—H1N	117 (2)	N2—C12—H12	117.9
C12—N2—C8	117.7 (2)	C11—C12—H12	117.9
C20—N3—C13	128.0 (2)	C14—C13—C18	118.2 (3)
C20—N3—H3N	131.8 (19)	C14—C13—N3	122.2 (3)
C13—N3—H3N	100.2 (19)	C18—C13—N3	119.5 (2)
C20—N4—C24	119.2 (2)	C15—C14—C13	120.1 (3)
C2—C1—C6	118.4 (2)	C15—C14—H14	119.9
C2—C1—N1	123.1 (2)	C13—C14—H14	119.9
C6—C1—N1	118.4 (2)	C14—C15—C16	122.3 (3)
C3—C2—C1	120.3 (2)	C14—C15—H15	118.8
C3—C2—H2	119.9	C16—C15—H15	118.8
C1—C2—H2	119.9	C17—C16—C15	117.7 (3)
C2—C3—C4	122.0 (2)	C17—C16—C19	121.4 (3)
C2—C3—H3	119.0	C15—C16—C19	121.0 (3)
C4—C3—H3	119.0	C16—C17—C18	120.5 (3)
C5—C4—C3	117.2 (2)	C16—C17—H17	119.8
C5—C4—C7	121.3 (2)	C18—C17—H17	119.8
C3—C4—C7	121.5 (3)	C17—C18—C13	121.2 (3)
C6—C5—C4	121.5 (2)	C17—C18—H18	119.4
C6—C5—H5	119.3	C13—C18—H18	119.4
C4—C5—H5	119.3	C16—C19—H19A	109.5
C5—C6—C1	120.6 (2)	C16—C19—H19B	109.5
C5—C6—H0	119.7	H19A—C19—H19B	109.5
C1—C6—H0	119.7	C16—C19—H19C	109.5
C4—C7—H7A	109.5	H19A—C19—H19C	109.5
C4—C7—H7B	109.5	H19B—C19—H19C	109.5
H7A—C7—H7B	109.5	N4—C20—N3	114.9 (2)
C4—C7—H7C	109.5	N4—C20—C21	121.7 (3)
H7A—C7—H7C	109.5	N3—C20—C21	123.4 (3)
H7B—C7—H7C	109.5	C22—C21—C20	118.0 (3)
N2—C8—N1	114.4 (2)	C22—C21—H21	121.0
N2—C8—C9	121.7 (2)	C20—C21—H21	121.0
N1—C8—C9	123.8 (2)	C23—C22—C21	121.3 (3)
C10—C9—C8	118.6 (3)	C23—C22—H22	119.4
C10—C9—H9	120.7	C21—C22—H22	119.4
C8—C9—H9	120.7	C22—C23—C24	117.1 (3)
C9—C10—C11	120.3 (3)	C22—C23—H23	121.4
C9—C10—H10	119.9	C24—C23—H23	121.4
C11—C10—H10	119.9	N4—C24—C23	122.7 (3)
C12—C11—C10	117.6 (2)	N4—C24—H24	118.7
C12—C11—H11	121.2	C23—C24—H24	118.7
			
C8—N1—C1—C2	39.0 (4)	C20—N3—C13—C14	−48.6 (4)
C8—N1—C1—C6	−144.6 (2)	C20—N3—C13—C18	135.1 (3)
C6—C1—C2—C3	1.5 (4)	C18—C13—C14—C15	−2.0 (4)
N1—C1—C2—C3	177.9 (2)	N3—C13—C14—C15	−178.4 (2)
C1—C2—C3—C4	−0.7 (4)	C13—C14—C15—C16	1.1 (4)
C2—C3—C4—C5	−1.0 (4)	C14—C15—C16—C17	0.7 (4)
C2—C3—C4—C7	178.3 (2)	C14—C15—C16—C19	−179.9 (3)
C3—C4—C5—C6	1.9 (4)	C15—C16—C17—C18	−1.4 (4)
C7—C4—C5—C6	−177.4 (2)	C19—C16—C17—C18	179.2 (2)
C4—C5—C6—C1	−1.1 (4)	C16—C17—C18—C13	0.4 (4)
C2—C1—C6—C5	−0.6 (4)	C14—C13—C18—C17	1.3 (4)
N1—C1—C6—C5	−177.2 (2)	N3—C13—C18—C17	177.8 (2)
C12—N2—C8—N1	−177.6 (2)	C24—N4—C20—N3	177.5 (2)
C12—N2—C8—C9	−0.2 (3)	C24—N4—C20—C21	−0.7 (4)
C1—N1—C8—N2	−166.9 (2)	C13—N3—C20—N4	176.3 (2)
C1—N1—C8—C9	15.8 (4)	C13—N3—C20—C21	−5.5 (4)
N2—C8—C9—C10	0.7 (4)	N4—C20—C21—C22	0.5 (4)
N1—C8—C9—C10	177.8 (2)	N3—C20—C21—C22	−177.5 (2)
C8—C9—C10—C11	−0.3 (4)	C20—C21—C22—C23	0.1 (4)
C9—C10—C11—C12	−0.4 (4)	C21—C22—C23—C24	−0.6 (4)
C8—N2—C12—C11	−0.6 (4)	C20—N4—C24—C23	0.2 (4)
C10—C11—C12—N2	0.9 (4)	C22—C23—C24—N4	0.5 (4)

### Hydrogen-bond geometry (Å, °)

**Table:**

*D*—H···*A*	*D*—H	H···*A*	*D*···*A*	*D*—H···*A*
N1—H1n···N2^i^	0.88 (1)	2.06 (1)	2.944 (3)	174 (3)
N3—H3n···N4^ii^	0.91 (1)	2.08 (2)	2.949 (3)	159 (3)

Symmetry codes: (i) −*x*, −*y*+1, −*z*; (ii) −*x*+1, −*y*+1, −*z*+1.

## References

[bb1] Barbour, L. J. (2001). *J. Supramol. Chem.* **1**, 189–191.

[bb2] Bruker (2007). *APEX2* and *SAINT* Bruker AXS Inc., Madison, Wisconsin, USA.

[bb3] Polamo, M., Repo, T. & Leskela, M. (1997). *Acta Chem. Scand.* **51**, 325–329.

[bb4] Sheldrick, G. M. (2008). *Acta Cryst.* A**64**, 112–122. 10.1107/S0108767307043930 18156677

[bb5] Westrip, S. P. (2008). *publCIF* In preparation.

